# Narrative discourse in Persian-speaking patients with mild Alzheimer’s disease

**DOI:** 10.1590/1980-57642018dn13-020012

**Published:** 2019

**Authors:** Masumeh Farivar, Zahra Ghayoumi Anaraki, Fatemeh Derakhshandeh, Nahid Baharloei, Marziyeh Poorjavad

**Affiliations:** 1 (MSc Student), Speech Therapy, Department of Speech Therapy, School of Rehabilitation Sciences, Isfahan University of Medical Sciences, Isfahan, Iran.; 2 (PhD), Speech Therapy, Department of Speech Therapy, School of Paramedical Sciences, Mashhad University of Medical Sciences, Mashhad, Iran.; 3 (PhD), Speech Therapy, Craniofacial and Cleft Research Center, Isfahan University of medical sciences, Isfahan, Iran.; 4 (MSc), Speech Therapy, Department of Speech Therapy, School of Rehabilitation Sciences, Isfahan University of Medical Sciences, Isfahan, Iran.; *5 PhD, Speech Therapy, Department of Speech Therapy, School of Rehabilitation Sciences, Isfahan University of Medical Sciences, Isfahan, Iran.

**Keywords:** oral narrative production, Alzheimer’s disease, aging, dementia, produção narrativa oral, doença de Alzheimer, envelhecimento, demência

## Abstract

Despite the significance of discourse impairments, they have not been thoroughly investigated in Persian-speaking patients with Alzheimer’s disease (AD).

**Objective:**

the aim of this study was to perform a multi-level analysis of narrative discourse in Persian-speaking patients with mild AD and to compare them with healthy elderly.

**Methods:**

the study included 14 older adults with mild AD and a matched group of 14 healthy elderly. Using a storytelling task based on serial pictures, both macro- and micro-linguistic aspects of narrative discourse were assessed. Cohesion ratio and coherence were investigated as macrolinguistic dimensions of discourse. The studied microlinguistic features included syntactic complexity and verbal errors (mostly involving phonological and semantic paraphasias and mazes). Severity of AD was determined using the Cognitive Dementia Rating (CDR).

**Results:**

there were significant differences between the groups regarding cohesion ratio (0.9 ± 0.34 vs. 1.29 ± 0.45, p = 0.02) and coherence scores (2.43 ± 0.41 vs. 3.02 ± 0.81, p = 0.03). Verbal errors and syntactic complexity did not differ significantly between the groups.

**Conclusion:**

Persian-speaking patients with mild AD show macrolinguistic impairments in producing discourses based on picture description. Therefore, intervention protocols should focus on the ability to organize information on a specific subject and also to connect sentences produced using appropriate cohesive ties.

## RESUMO.

Apesar da significância das deficiências discursivas, elas não foram completamente investigadas em pacientes de fala persa com doença de Alzheimer (DA).

**Objetivo:**

o objetivo deste estudo foi desenvolver uma análise multinível do discurso narrativo em pacientes de língua persa com DA leve e compará-los com idosos saudáveis.

**Métodos:**

o estudo incluiu 14 idosos com DA leve e um grupo pareado de 14 idosos saudáveis. Usando uma tarefa narrativa baseada em imagens seriais, os aspectos macro e microlinguísticos do discurso narrativo foram avaliados. Coesão e coesão foram investigadas como dimensões macrolinguísticas do discurso. As características microlinguísticas estudadas incluíram complexidade sintática e erros verbais (incluindo principalmente parafasias e labirintos fonológicos e semânticos). A gravidade da DA foi avaliada por meio do Cognitive Dementia Rating (CDR).

**Resultados:**

houve diferenças significativas entre os grupos quanto ao coeficiente de correlação (0,9 ± 0,34 vs. 1,29 ± 0,45, p = 0,02) e escores de coerência (2,43 ± 0,41 vs. 3,02 ± 0,81, p = 0,03). Erros verbais e complexidade sintática não foram significativamente diferentes entre os grupos.

**Conclusão:**

pacientes de língua persa com DA leve apresentam comprometimento macrolinguístico em produzir discursos baseados na descrição de figuras. Portanto, os protocolos de intervenção devem enfocar sua capacidade de organizar informações sobre um assunto específico e também conectar suas sentenças produzidas por laços coesos apropriados.

The rise in the elderly population has posed social, economic and health challenges for the twenty-first century. According to the World Health Organization (WHO), the proportion of the population aged over 60 is increasing dramatically worldwide.^1^ Older age is usually accompanied by decline in physiological and physical performance.^2^ Alzheimer’s disease (AD), a prevalent condition in the elderly, leads to chronic impairments in cognition, memory, thinking and language.^3^ Language dysfunction is observed across all stages of AD and is one of the clinical symptoms for diagnosing the disease.^4^^,^ 5

Attention to the language dysfunction is of immense importance as it dramatically affects patients’ communication with others. A number of studies^6^^-^ 9 have shown that some deficits in discourse cause communication breakdowns in AD patients. Discourse is a unit of language whose components are interconnected and fulfil a specific communicative purpose. This complex level of language is beyond the level of phrases and sentences and involves different elements of the language system (phonological, morphological, lexical, semantic and syntactic levels), as well as other cognitive areas such as executive functions.^10^^,^ 11 This complex performance has been shown to require interaction between the prefrontal cortex, and left anterior and bilateral posterior perisylvian regions.^6^^,^ 12 ^,^ 13

Efficient discourse involves diverse micro- and macrolinguistic dimensions. Lima, Brandão, Parente and Peña-Casanova^8^ investigated coherence as a macrolinguistic skill in Spanish-speaking patients with moderate AD. Coherence indicates the topic integrity of the discourse and demonstrates the relationship between the content or meaning of an utterance and the general topic.^14^ Understanding of a coherent discourse is easy since its parts are connected in a clear and logical way. Lima et al.^8^ revealed that patients with AD produced significantly less coherent discourse in comparison with healthy elderly. Drummond et al.^7^ also showed that discourses produced by patients with AD was significantly weaker in terms of general coherence and referential cohesion than that produced by healthy elderly individuals and also those with mild cognitive impairments.

Although deficits in macrolinguistic dimensions of discourse have been reported in patients with mild AD by the majority of previous studies,^7^^,^ 8 ^,^ 15 some studies have revealed deficits in microlinguistic aspects such as semantic and syntactic levels.^6^^,^ 9 Choi,^6^ for instance, demonstrated that although syntactic aspects were preserved in the early stages of AD, some impairments in semantic aspects of the language affected discourses produced by patients with mild AD.^6^

As mentioned above, discourse involves various dimensions. The previously published studies have considered different sets of measures to study narrative discourse in patients with AD. This heterogeneity in assessment methods hampers comparison of study results. Moreover, a multi-level discourse analysis (both macro- and micro-linguistic levels), compared to the analysis of individual aspects, has been shown to offer a more realistic perspective of the discourse.^16^ In addition, despite the significance of discourse impairments in communication, they have not been investigated thoroughly in Persian-speaking patients with AD. Each language involves a unique system of rules and features which govern communication, and the results of studies on discourse may be affected by differences in the languages studied. For instance, Persian in contrast to English, is a pro-drop language.^17^ It also has stronger conjugation, where verbs change according to the sentence’s subject.^11^ Therefore, investigating discourse features of the Persian language, which might be affected by AD, can be of great help to speech and language therapists in better handling patients with AD to achieve more effective communication. Accordingly, the aim of this study was to develop a multi-level analysis of narrative discourse in Persian- speaking patients with mild AD and to compare them with healthy elderly individuals in order to provide more effective rehabilitation interventions.

## METHODS

Fourteen elderly individuals with mild AD and fourteen healthy elderly individuals, who attended an elderly health care center, were selected for participation in this study. At the center, usual medical services for elderly people were provided by geriatric physicians. The healthy participants were matched with the AD group according to age, gender, and level of education. AD patients were aged 60 years or older. Based on neuroimaging, laboratory, and neuropsychiatric tests, all patients were diagnosed by neurologists as having definite AD. The patients had no history of stroke, traumatic brain injury or other known neurological, neuropsychiatric and neuropsychological disorders. The patients were also monolingual and literate in Persian. In order to rule out the likelihood of aphasic language disorders, the Persian version of the Western Aphasia Battery (P-WAB) was used. This is a bedside version of the Western Aphasia Battery (WAB) used as a quick valid clinical screening of aphasia in Persian-speaking brain-damaged patients. The P-WAB consists of 6 subtests including spontaneous speech content, fluency of spontaneous speech, auditory comprehension, sequential commands, repetition and naming. Each subtest is scored out of 10 and then a percentile Aphasia Quotient (AQ) is calculated based on the row scores to determine the severity of aphasia (mild, moderate, severe, or very severe).^18^ An AQ score of 91 or more has been reported to indicate that the person should not be considered aphasic.^11^ Therefore, patients who scored 91 or more on the P-WAB were included. The healthy elderly individuals were also aged 60 years or older and had no history of stroke, traumatic brain injury, or other known neurological, neuropsychiatric and neuropsychological disorders. The healthy subjects were also monolingual and literate in Persian. Participants that had significant hearing or visual impairments which affected their ability to respond were excluded from the study. The study was approved by the Medical Research Ethics Committee of Isfahan University of Medical Sciences and all participants or their families provided informed consent.

To investigate the cognitive functioning of the participants, the validated Persian version of the Clinical Dementia Rating (CDR)^19^^,^ 20 was individually administered by a geriatric physician. The CDR is a reliable instrument for staging Alzheimer’s disease severity. This scale consists of 75 questions in 6 domains including memory, time and space orientation, judgment and problem solving, social affairs, home and hobbies, and personal care. Each domain (except the personal care domain) is scored from 0 to 3 as follows: 0, no impairment; 0.5, questionable impairment; 1, mild impairment; 2, moderate impairment; and 3, severe impairment. A global CDR score can be obtained based on the scores of each domain.^21^ The current study included patients with mild AD (CDR score = 0.5-1) and healthy elderly individuals with normal cognition (CDR score = 0).

The narrative discourse abilities in both groups were assessed using a storytelling task.^11^ The task includes six serial pictures, all on the same page, with a familiar topic to Iranian subjects. The topic consists of an initiating event that prompts a character to act, an attempt related to the initiating event, and a direct consequence of the attempt. The pictures were shown to the participants in an individual assessment session. They were asked to narrate a story concerning the events illustrated in the pictures. No time limit was imposed for the test; the pictures remained in front of the participants until the end of the task in order to avoid poor performance due to memory constraints. No additional cues or tips were provided to assist the participants.^11^ The narrations were audio-recorded and coded for further blind analysis by two researchers. The cohesion and coherence ratios, as the macrolinguistic dimensions of the discourse, along with the sentence complexity and verbal errors ratio, as the microlinguistic dimensions of the discourse, were extracted. The number of communication units (C-units) was obtained in each discourse sample, according to the method proposed by Ghayoumi Anaraki et al.^11^ A C- unit is an independent clause with all attached subordinate clauses. Other linguistic measures were then calculated based on the C-units produced. Different cohesive ties, including substitutions, ellipses, conjunctions, references and lexical markers, were analyzed. The total number of cohesive ties produced was divided by the total number of C-units produced to yield the cohesion ratio. As mentioned earlier, coherence as one of the macrolinguistic dimensions indicates the topic integrity of the discourse. Each C-unit produced was scored between 1 and 4 based on the extent to which it related to the overall topic. The mean of coherence scores was then computed for each narrative sample to give global coherence. On this 4- point scale, a score of 1 indicates that the C-unit produced is completely unrelated to the topic, whereas a score of 4 indicates C-units that include significant details of the stimulus and thus, are overtly related to the topic. The verbal errors ratio was calculated by determining the total number of errors and then dividing this by the total number of C-units. These errors are generally phonological and semantic paraphasias, neologisms, and mazes.^11^^,^ 22 Mazes are linguistic disfluencies including a series of words or parts of words which do not alter the meaning of the C-units. They can be filled pauses, repetitions or revisions.^23^ Filled pauses are vocalizations like ‘um’ and ‘uh’ that usually signal upcoming delays.^24^ Repetitions occur when the subject immediately repeats a word or a phrase.^25^ Revisions include the subject’s corrections of the words or ideas.^9^ These kinds of disfluencies usually occur when individuals are speaking about ideas that are abstract and/or complicated.^26^ The sentence complexity in this task is the syntactic complexity, as an index of language proficiency.^27^A complex sentence is a sentence with an independent clause and at least one dependent clause. The syntactic complexity measure in this study was obtained by determining the number of all dependent and independent clauses and dividing these by the total number of C-units.^11^

Statistical analyses were performed using SPSS version 20 (version 20, SPSS Inc., Chicago, IL). The Kolmogorov-Smirnov test was used to evaluate the normality of the distribution of variables. The mean of variables with normal distribution (cohesion ratio, coherence scores, sentence complexity and age) were then compared between the two groups using independent T-tests. A Mann-Whitney U-test was carried out to examine differences between the two groups for verbal error ratio. The differences between the groups were compared using the Chi-square test for nominal variables, including gender and level of education.

## RESULTS

The mean age of the AD patients (66.85 ± 4.63 years) did not differ significantly from that of the healthy individuals (64.28 ± 3.36 years) (P = 0.11). Table 1 shows demographic characteristics of the participants. There were no significant differences between the groups in terms of gender (p = 1) or level of education (P = 0.70).

**Table 1:** Demographic information for patients with Alzheimer’s disease and healthy elderly subjects.

		AD patients	Healthy elderly subjects	P _value_
Age mean (SD)		66.85 (4.63)	64.28 (3.36)	0.11
Gender	Female n (%)	10 (28.6%)	10 (28.6%)	1
	Male n (%)	4 (71.4%)	4 (71.4%)	
Education level	Not a high school graduate n (%)	3 (21.45%)	5 (35.7%)	0.70
	High school graduate or associate degree n (%)	6 (42.85%)	5 (35.7%)	
	Bachelor’s degree or higher n (%)	5 (35.7%)	4 (28.6%)	

AD: Alzheimer’s disease; SD: standard deviation; n: number.

The means of the cohesion ratio and coherence scores in the healthy individuals were significantly higher than those of the AD patients. Concerning the verbal errors and sentence complexity, the AD group produced, on average, less complex sentences, together with more verbal errors, in comparison to the healthy individuals, although the differences between the two groups were not statistically significant (Table 2).

**Table 2:** Narrative discourse features in patients with Alzheimer’s disease and healthy elderly subjects.

	AD patients Mean (SD)	Healthy elderly subjects Mean (SD)	P _value_
Sentence complexity	1.29 (0.28)	1.44 (0.29)	0.17
Verbal error ratio	0.51 (0.42)	0.35 (0.22)^*^	0.32
Cohesion ratio	0.91 (0.34)	1.29 (0.45)	0.02
Coherence	2.43 (0.41)	3.02 (0.81)	0.03

AD: Alzheimer’s disease; SD: standard deviation;

* N = 13, Due to missing verbal error ratio data for one healthy subject.

## DISCUSSION

The aim of this study was to perform a multi-level analysis of narrative discourse in Persian-speaking patients with mild AD and to compare them with healthy elderly individuals. Using a storytelling task based on serial pictures, both macro- and micro-linguistic aspects of narrative discourse were assessed. Cohesion ratio and coherence were investigated as macrolinguistic dimensions of discourse. The microlinguistic features studied included syntactic complexity and verbal errors. Overall, results revealed that the patients with mild AD had significantly poorer performance than the healthy subjects for macrolinguistic features of discourse. This finding confirmed that AD, even in its early stages, can disrupt communication through detrimental effects on discourse macrolinguistic dimensions in the Persian language. On the other hand, microlinguistic aspects of narrative discourse, i.e. verbal errors and sentence complexity, seem to be relatively preserved, at least during the early stages of the disease. These findings have implications for speech and language therapists seeking to provide effective intervention protocols for enhancing communication efficiency of Persian-speaking patients with mild AD.

Coherence scores indicate an individual’s ability to organize and integrate intratextual information of a discourse.^11^ The proper organization of information in a way that allows the listener to interpret the meaning of discourse requires relatively intact access to semantic memory representations of surrounding real-world items and concepts. Also, the speaker needs to recruit simultaneous attention and mental manipulation of extensive information so they can organize the information in a way that the listener comprehends the speech from his/her point of view and based on his/her own knowledge.^28^ Consistent with our results, decline in discourse coherence in AD patients has also been reported in other languages, including Brazilian Portuguese,^7^ Spanish^8^ and Chinese.^15^ Lai^15^ showed that Chinese-speaking patients with AD tended to produce less coherent discourses including more circumlocutionary comments compared with the control group. Lai^15^ concluded that this language behavior can possibly be used as a coping strategy to overcome the patient’s inability to examine and pay attention to the context and setting of communication. In the Spanish language, Lima and colleagues^8^ also observed similar impairments in patients with moderate and moderate-severe cognitive decline caused by AD. Their patients had significant difficulties in expressing knowledge and these discourse impairments were strongly related to the severity of cognitive decline. Drummond et al.^7^ also reported less coherent discourses in mild AD patients than both the control group and patients with mild cognitive deficits. Based on the number of words produced by the AD patients, they concluded that coherence impairments cannot stem only from lexical-semantic deficits in these patients; rather their inability to organize information and episodes of a topic leads to a less coherent discourse.^7^

Global coherence has been shown to be closely related to working memory.^8^ In fact, to carry out such a complex task, individuals need to pay attention to the cues in the picture, while planning their verbal output and also keeping in mind what has already been expressed and what has yet to be expressed. In addition, as mentioned above, they have to direct their attention to the cues in the context of dialogues and the listener’s needs. It seems that the efficiency of the central executive system of working memory is affected in the early stages of AD for processing this complex task.^8^

Cohesive ties help individuals logically link their utterances so that the listener can follow them.^29^ Consistent with Drummond et al.^7^ and Carlomagno et al.,^30^ the present study showed that individuals with mild AD used less cohesive ties in their discourse. Only 28% of the mild AD patients in the study by Drummond et al.^7^ demonstrated an adequate pattern of cohesion in their discourse. Other patients committed many errors, such as incorrect deletion of pronoun reference and inappropriate or ambiguous use of pronouns. Working memory deficits, semantic-pragmatic difficulties, and lexical retrieval impairments during the production of discourse appeared to be the deficits that led to patterns of cohesion impairments.^7^^,^ 30

Our results indicated that, although the mild AD patients performed more poorly compared with the healthy elderly individuals with regard to sentence complexity, the difference between the two groups was not statistically significant. Studies on other languages^6^^,^ 28 ^,^ 30 have also indicated, at least during the early stages of AD, that the phonological and syntactic aspects of the language are relatively preserved. Choi^6^ showed that discourses produced by a group of Japanese patients with mild AD were inefficient and empty in semantic aspects. The patients also performed worse than the healthy elderly in confrontation naming tests. By contrast, all indices of syntactic aspects of narration were comparable between patients with mild AD and healthy elderly. Thus, this aspect of language seems not to be affected by early stages of the disease and, therefore, cannot be considered critical features distinguishing the language patterns of mild AD patients from those of normal individuals.^6^ Lai, Pai and Lin^25^ also analyzed picture descriptions provided by Chinese-speaking persons with mild to moderate dementia. Regarding the clause type, the AD patients produced significantly fewer dependent and independent clauses than the elderly control participants. Moreover, patients used certain complex constructions less frequently. They were, however, able to produce the entire variety of sentence types and did not make more syntactic errors compared with the healthy controls. Based on these findings, the authors concluded that the AD patients’ syntactic knowledge is relatively preserved.^25^

The results of this study concerning verbal errors indicated no significant differences between the two groups. Lai et al.^25^ also indicated there were no significant differences between patients with mild-to-moderate AD and healthy controls regarding the production of repetitions and semantic substitutions. However, de Lira et al.,^9^ who investigated microlinguistic aspects in depth, including syntactic complexity and some types of verbal errors, showed significant differences between the healthy elderly and AD patients. They showed that the AD patients produced significantly simpler syntactic utterances and more lexical errors (including word repetitions and revisions, but not phonemic paraphasias) in their oral discourses compared with healthy elderly controls. A multiple logistic regression analysis identified repetitions, revisions and coordinated syntactic sentences as the variables which could differentiate AD subjects from the healthy controls, independent of age. These authors suggested that close attention to these features could help propose other underlying pathologies for communication impairments caused by the disease.^9^ In contrast, Lai^15^ showed that patients with different severity levels of AD made significantly fewer revisions than the control group in their discourses. Since “revision”, as a higher-level processing function, requires self- monitoring of errors and production of alternatives, Lai^15^ concluded that the AD patients might fail to successfully revise the errors produced. Nevertheless, it should be noted that severity of the disease, and methods used for evaluating syntactic features and verbal errors are also of paramount importance in drawing conclusions from different studies. For instance, the sentence complexity in the current study was not analyzed in the manner investigated in the studies by Lai et al.^25^ or de Lira et al..^9^ Also, although different subtypes of verbal errors in this study (i.e. revisions, repetitions, filled pauses, and paraphasias) were similar to other studies,^9^^,^ 15 ^,^ 25 we reported a global score for these, as opposed to analyzing them individually.

There were a number of limitations in the current study which need to be acknowledged. Although, to the best of our knowledge, this kind of comprehensive and multi-level analysis of narrative discourse has not been done before in AD patients with the Persian language, we acknowledge that a relatively small sample of subjects were included in this study. Therefore, future studies with a larger sample size may be needed to confirm the findings. Moreover, the level of education of the speakers can partially affect different narrative discourse features.^31^ Thus, another limitations of this study may be related to the classification and matching of the participants in terms of their level of education. In this broad classification, each educational level includes persons with a different number of years of education. Therefore, if the participants had been matched based on the exact number of years of education, perhaps differences caused only by their cognitive status could have been better shown. Future studies could also investigate the influence of features other than severity of cognitive impairment, such as educational level and socioeconomic status, on the language profile of the AD patients.

In conclusion, our findings demonstrated that Persian-speaking patients with mild AD exhibit macrolinguistic impairments in producing discourses based on pictures description. These patients tend to produce less coherent oral narratives compared to the healthy elderly. This means Alzheimer’s disease, even in early stages, may impair the patient’s ability to organize and manipulate information regarding a specific subject. Moreover, despite comparable syntactic complexity, patients used less cohesive ties than the healthy older adults. This implies that their communication partners are less likely to be able to follow the oral narratives produced. Therefore, in order to enhance communication efficiency of these patients, intervention protocols should focus on their ability to organize information regarding a specific subject, to make sentences that are related to the overall topic of the discourse, and also to connect the sentences produced using appropriate cohesive ties.
